# Declining Outcrossing Rates Inside Orchard Blocks of ‘Maluma’ and ‘Shepard’ Avocado (*Persea americana* Mill.) Trees: Effects on Fruit Yield and Quality

**DOI:** 10.3390/plants14081218

**Published:** 2025-04-15

**Authors:** Matthias A. Reese, Rachele S. Wilson, Joel Nichols, Stephen J. Trueman

**Affiliations:** School of Environment and Science, Griffith University, Brisbane South (Nathan) Campus, Brisbane, QLD 4111, Australia; matthias.reese@griffithuni.edu.au (M.A.R.); rachele.wilson@griffith.edu.au (R.S.W.); j.nichols2@griffith.edu.au (J.N.)

**Keywords:** cross-fertilisation, cross-pollination, mating system, *Persea americana*, polliniser, self-compatibility

## Abstract

Many rapidly expanding food crops, including avocado (*Persea americana* Mill.), are dependent on animal pollination but there is a growing shortfall in global pollinator supply. Avocado flowers are insect-pollinated and yields of the main cultivar, ‘Hass’, are often pollen-limited, especially in the middle of single-cultivar orchard blocks, where there is limited deposition of cross-pollen from another cultivar. We analysed two avocado cultivars of alternate flowering types, ‘Maluma’ (Type A) and ‘Shepard’ (Type B), using SNP-based DNA markers to identify the pollen parent of fruit at different distances from the other cultivar. We aimed to determine whether the numbers of cross-fertilised fruit and tree yields decline at increasing distances from a cross-pollen source, and whether cross-fertilised fruit are larger than self-fertilised fruit. We found that the number of cross-fertilised fruit produced by each tree declined in the middle of the blocks of each cultivar. Trees in the middle of the ‘Maluma’ block compensated for low levels of cross-pollination by producing more self-fertilised fruit, and their yields did not appear to be pollen-limited. However, yields in the middle of the ‘Shepard’ block declined by 25% as a direct result of a 43% reduction in the number of cross-fertilised fruit produced by each tree. ‘Shepard’ trees did not compensate for poor cross-pollination by producing more self-fertilised fruit. Cross-fertilisation of ‘Maluma’ by ‘Shepard’ increased fruit mass by 8% and cross-fertilisation of ‘Shepard’ by ‘Hass’ increased fruit mass by 5%, compared with self-fertilisation. Our results confirm that yields of avocado trees are sometimes, but not always, pollen-limited. Low levels of both self-pollination and cross-pollination resulted in pollen limitation of yield in the middle of the ‘Shepard’ block, but high levels of self-pollination were sufficient to generate high yields in the middle of the ‘Maluma’ block. Closer interplanting of Type A and Type B avocado cultivars increases the opportunities for cross-pollination, which can often increase tree yield and fruit size, and improve the financial returns for growers. Improving the pollination efficiency of foraging insects by providing them with the optimal pollen genotypes is increasingly important as we experience a growing demand for managed pollinators and a declining abundance of wild pollinators.

## 1. Introduction

The global demand for food is expected to increase by 35–62% in the 40-year period to 2050 [1]. Fruit crops are providing a steadily increasing contribution to global food security and human nutrition, generating around 910 million tons, or about 9%, of the current annual food production [2]. However, yields fluctuate greatly due to factors that include climate variability, flooding and drought [3,4,5,6,7], suboptimal nutrient availability [7,8,9], and growing deficits in the abundance and diversity of pollinators [10,11,12,13,14,15]. The demand for pollination services in agriculture is growing because of rapid expansion in the cultivated area of pollinator-dependent crops [16]. Most of the fastest-expanding crops, such as avocado (*Persea americana* Mill.), citrus (*Citrus* L. spp.), plum (*Prunus* L. spp.), okra (*Abelmoschus esculentus* Moench), cashew (*Anacardium occidentale* L.), chestnut (*Castanea sativa* Mill.), and palm oil (*Elaeis guineensis* Jacq.), are pollinator-dependent to various degrees, while the cultivated area is decreasing for many crops that are not pollinator-dependent, such as barley (*Hordeum vulgare* L.), oats (*Avena sativa* L.), sorghum [*Sorghum bicolor* (L.) Moench], potato (*Solanum tuberosum* L.), sweet potato [*Ipomoea batatas* (L.) Lam.], and sugar beet (*Beta vulgaris* L.) [16,17]. Pollinator-dependent crops are major providers of many critical human nutrients and antioxidants such as vitamins A, C, and E, carotenes, tocopherols, and lycopene [18]. Global food supply and human nutrition are, therefore, increasingly dependent on ensuring highly efficient crop pollination despite a growing shortfall in pollinator supply [14,19,20,21].

Many animal-pollinated fruit and nut crops are self-incompatible, requiring that the pollinators deposit pollen from a different genotype on the stigma to ensure fertilisation and fruit production [22,23,24,25,26]. Many other fruit and nut crops are self-compatible, being capable of setting fruit after self-pollination by the same genotype, although they often produce a mixture of self-fertilised and cross-fertilised fruit in commercial orchards [26,27,28,29,30,31,32]. Many crops selectively retain cross-fertilised fruitlets rather than self-fertilised fruitlets during the period of early fruit development and premature fruit drop [33,34,35,36,37,38,39,40], and cross-fertilised fruit are often larger than self-fertilised fruit [25,27,34,41,42,43,44]. Therefore, yields are often higher in orchards where the levels of cross-pollination are maximised to ensure high levels of outcrossing [45,46,47,48]. Furthermore, cross-fertilised fruit sometimes have better taste attributes, skin colour, nutrient content, or shelf life than self-fertilised fruit [32,41,49,50,51,52]. The effects of different pollen parents, including cross-paternity versus self-paternity, on the size, quality, and other parameters of the fruit and seeds are known as xenia [53].

Avocado is a rapidly expanding tropical tree crop that produces fruit with a highly nutritious fatty-acid profile [54,55,56,57]. Avocado trees are grown in almost 100 countries, with the top 10 producers being Mexico, Colombia, Peru, Dominican Republic, Kenya, Indonesia, Brazil, Vietnam, Israel, and Haiti [58]. Avocado flowers are pollinated by bees and other insects [59,60,61,62] and the realised mating system is mixed-mating, with avocado trees in orchards typically producing a mixture of self-fertilised and cross-fertilised fruit [33,35,38,63,64,65,66,67]. The predominant avocado cultivar globally is ‘Hass’, but orchards are usually established with additional cultivars to maximise the opportunities for successful pollen deposition on the stigmas of flowers [33,38,65,66,67]. The stigmas of most avocado flowers receive few or no pollen grains, partly because there is limited overlap during each day between the opening of the female phase and the male phase of flowers within each cultivar [65,68,69,70]. The flowers of Type A cultivars such as ‘Hass’ and ‘Maluma’ open in the female phase in the morning, close in the afternoon, and re-open in the male phase on the afternoon of the following day [71,72,73,74,75]. The flowers of Type B cultivars such as ‘Fuerte’ and ‘Shepard’ open in the female phase for the afternoon of one day, close overnight, and re-open in the male phase for the morning of the following day [71,72,73,74,75]. This means that the opportunities for effective pollen deposition on the stigma of female-phase flowers may be greatest when they have ready access to the open male-phase flowers of an alternate-type cultivar [67,71,74]. However, many orchards are planted predominantly with the most desired cultivar (e.g., ‘Hass’) or they are established with the different cultivars each planted in wide blocks so that the management of pests, diseases, fertilisers, irrigation, harvesting, and post-harvest processing can cater conveniently and cost-effectively to individual cultivars [38,64,76,77,78]. This type of orchard design can reduce the opportunities for effective pollen deposition, especially in the middle of the wide blocks where pollinators may need to forage across many rows of trees to transfer pollen from a Type B cultivar to a Type A cultivar, or vice versa.

Outcrossing levels often decline at increasing distances from another cultivar in avocado orchards [33,35,38,63,64,66,67]. The declining outcrossing levels have sometimes been related to declining yields [33,35,64,67]. We have shown recently that declining outcrossing levels in the middle of a wide block of ‘Hass’ trees were the basis for a 44% decline in yield, caused directly by a 69% reduction in the number of cross-fertilised fruit per tree without a significant reduction in the number of self-fertilised fruit per tree [67]. This indicates that there was an underlying level of self-pollination throughout the block but there was limited transport of cross-pollen by insects into the middle of the block. Furthermore, we found that the cross-fertilised ‘Hass’ fruit (which were pollinated by ‘Shepard’) were 12% heavier than self-fertilised ‘Hass’ fruit [67], similar to the differences found previously between ‘Hass’ fruit that were cross-fertilised by ‘Fuerte’ and self-fertilised ‘Hass’ fruit [69]. These results demonstrated empirically that improved proximity to the open male-phase flowers of a Type B cultivar increases the opportunities for cross-pollination of ‘Hass’ (Type A) flowers and that this can result in more fruit per tree, greater mass of individual fruit, and higher tree yield. The extent to which closer proximity to cross-pollen sources and increasing cross-pollination would affect the number of outcrossed fruit per tree, individual fruit mass, and tree yield remains largely unknown for many other avocado cultivars.

In this study, we asked whether closer proximity to cross-pollen sources would affect outcrossing levels, the number of outcrossed fruit per tree, individual fruit mass, and tree yield in another Type A cultivar (‘Maluma’) and a Type B cultivar (‘Shepard’). The purple-skinned cultivar, ‘Hass’, is the most widely grown avocado cultivar globally but the green-skinned cultivar, ‘Shepard’, is often interplanted as a polliniser for ‘Hass’, partly because it has a prolonged flowering period [79]. The purple-skinned cultivar, ‘Maluma’, is increasingly grown as an alternative to ‘Hass’ because its fruit mature earlier in the harvest season than ‘Hass’ and it appears to be an effective polliniser for ‘Shepard’ during the early part of the flowering period [79]. We hypothesised that (a) outcrossing levels among the fruit of ‘Maluma’ and ‘Shepard’ trees would decline at increasing distances from blocks of trees of the alternate flowering type; (b) the number of cross-fertilised fruit per tree would decline at increasing distances from blocks of trees of the other cultivar; (c) tree yields would also decline at increasing distances from the other cultivar; and (d) cross-fertilised fruit would be larger than self-fertilised fruit. The results of this study will assist in designing avocado orchards for more efficient pollination, higher yields, and optimal fruit quality in the face of growing shortfalls in the availability and diversity of pollinating insects.

## 2. Results

**Figure 1:**
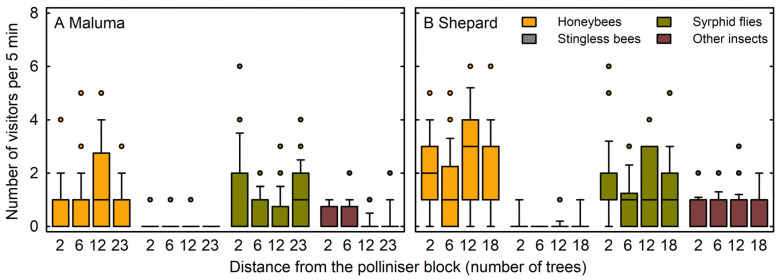
Numbers of honeybees (*Apis mellifera* L.), stingless bees (*Tetragonula* Moure spp.), syrphid flies (*Syrphidae latreille* spp.), and other insects visiting the flowers of (**A**) ‘Maluma’ and (**B**) ‘Shepard’ avocado (*Persea americana* Mill.) trees per 5 min period. Medians are presented with 25th and 75th percentiles (boxes), 10th and 90th percentiles (whiskers), and outliers (circles). The four medians and data distributions within each insect group for each avocado cultivar do not differ significantly (median and Kruskal–Wallis tests, respectively; *p* > 0.05; n = 24–28).

The predominant visitors to avocado flowers at the study site were honeybees (*Apis mellifera* L.) and syrphid flies (*Syrphidae latreille* spp.), with smaller numbers of other insects (Figure 1). Very few stingless bees (*Tetragonula* Moure spp.) were observed. The abundance of visitors from any of the four insect groups did not differ significantly among the four tree positions in the ‘Maluma’ block (Figure 1A) or the ‘Shepard’ block (Figure 1B). A total of 85% of honeybees on ‘Maluma’ flowers were foraging on nectar, while 84% of honeybees on ‘Shepard’ flowers were foraging on pollen. A total of 72% of ‘Maluma’ trees possessed only female-phase flowers at the time of observations, while the remaining 28% of ‘Maluma’ trees possessed both female-phase and male-phase flowers. Almost all ‘Shepard’ trees (99%) possessed only male-phase flowers at the time of observations.

**Figure 2:**
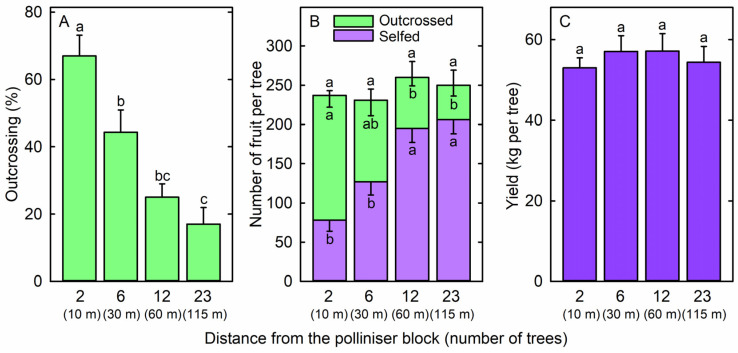
(**A**) Outcrossing rate among fruit, (**B**) number of outcrossed fruit, number of selfed fruit, and total number of fruit, and (**C**) fruit yield of ‘Maluma’ avocado (*Persea americana* Mill.) trees located at 2 trees (10 m), 6 trees (30 m), 12 trees (60 m), or 23 trees (135 m) from a block of ‘Shepard’ polliniser trees. Means (+SE for outcrossing rate, total number of fruit, and fruit yield; −SE for number of selfed fruit and number of outcrossed fruit) with different letters are significantly different (random block ANOVA and Tukey’s HSD test; *p* < 0.05; n = 10 trees).

The percentage of ‘Maluma’ fruit that were outcrossed declined progressively from 67 ± 6% at two trees away from cultivar ‘Shepard’ to 17 ± 5% at twenty-three trees away from cultivar ‘Shepard’ (Figure 2A). Almost all outcrossed ‘Maluma’ fruit were fertilised by ‘Shepard’, although six ‘Maluma’ fruit were fertilised by ‘Hass’. The number of outcrossed fruit per ‘Maluma’ tree decreased from 159 ± 15 at two trees away from ‘Shepard’ to 44 ± 14 at twenty-three trees away from ‘Shepard’ (Figure 2B). However, the number of selfed fruit per ‘Maluma’ tree increased from 78 ± 14 at two trees away from ‘Shepard’ to 206 ± 18 at twenty-three trees away from ‘Shepard’ (Figure 2B). As a result, the total number of fruit per tree did not differ significantly among the four positions within the ‘Maluma’ block (Figure 2B). Tree yield also did not differ significantly among the four positions within the ‘Maluma’ block, with yields being between 53.0 ± 2.5 kg and 57.2 ± 4.3 kg per tree (Figure 2C).

The ‘Maluma’ fruit that were fertilised by ‘Shepard’ had 2.0 mm greater diameter and 1.2 mm greater length than self-fertilised ‘Maluma’ fruit (Table 1). They were 8% heavier than the selfed fruit, with 10% heavier seeds and 8% heavier pericarp (Table 1).

**Figure 3:**
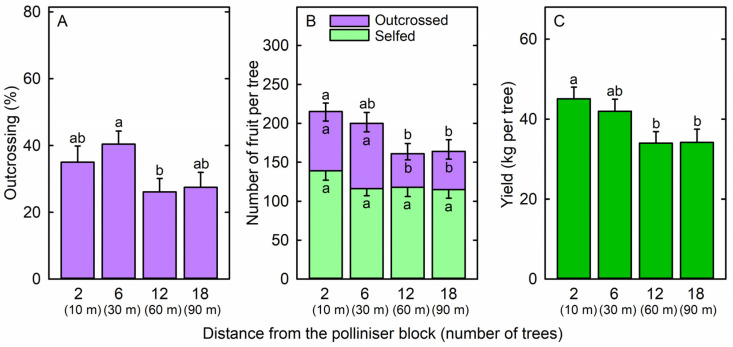
(**A**) Outcrossing rate among fruit, (**B**) number of outcrossed fruit, number of selfed fruit, and total number of fruit, and (**C**) fruit yield of ‘Shepard’ avocado (*Persea americana* Mill.) trees located at 2 trees (10 m), 6 trees (30 m), 12 trees (60 m), or 18 trees (90 m) from a block of ‘Maluma’ polliniser trees. Means (+SE for outcrossing rate, total number of fruit, and fruit yield; −SE for number of selfed fruit and number of outcrossed fruit) with different letters are significantly different (two-way ANOVA and Tukey’s HSD test; *p* < 0.05; n = 12 trees).

**Figure 4:**
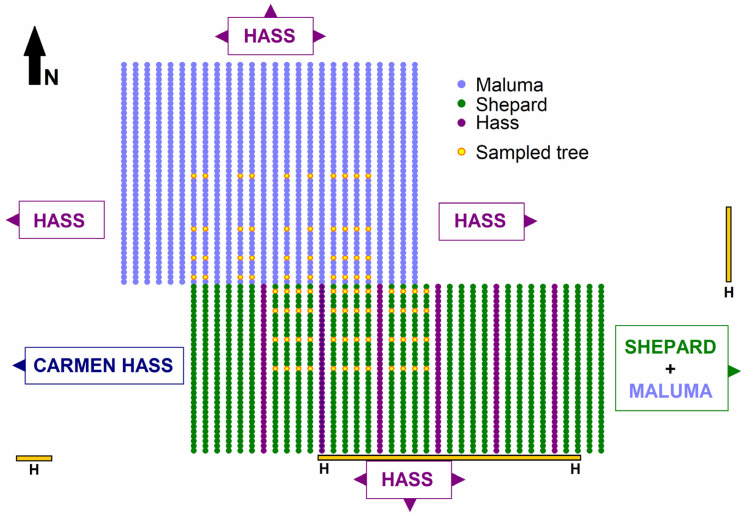
Schematic diagram of the avocado (*Persea americana* Mill.) orchard, showing the block of ‘Maluma’ trees and the block containing ‘Shepard’ and interplanted ‘Hass’ trees, with the 88 sampled trees shown as yellow dots. The positions of the adjoining blocks of ‘Hass’, ‘Carmen Hass’, and the mixture of ‘Shepard’ and interplanted ‘Maluma’ trees are indicated with arrows. The approximate locations of bee hives are marked with gold bars, labelled ‘H’.

The percentage of ‘Shepard’ fruit that were outcrossed declined from 40 ± 4% at six trees away from ‘Maluma’ to 26 ± 4% at twelve trees away from ‘Maluma’ but did not differ significantly among other positions in the ‘Shepard’ block (Figure 3A). The percentage of fruit that were fertilised by ‘Hass’ was 3 times the percentage that were fertilised by ‘Maluma’ in this ‘Shepard’ block, which was interplanted at every fifth row with ‘Hass’ trees (Figure 4). Nonetheless, the number of outcrossed fruit per ‘Shepard’ tree decreased from 76 ± 12 and 84 ± 11 at two and six trees, respectively, away from ‘Maluma’, to 43 ± 8 and 49 ± 10 at twelve and eighteen trees, respectively, away from ‘Maluma’ (Figure 3B). The number of selfed fruit per tree did not differ significantly among the four positions within the ‘Shepard’ block (Figure 3B). The total number of fruit per tree declined from 215 ± 11 at two trees away from ‘Maluma’ to 161 ± 13 and 164 ± 15 at twelve and eighteen trees, respectively, away from ‘Maluma’ (Figure 3B). Tree yield declined from 45.1 ± 2.9 kg at two trees away from ‘Maluma’ to 34.0 ± 2.9 kg and 34.2 ± 3.3 kg at twelve and eighteen trees, respectively, away from ‘Maluma’ (Figure 3C). The outcrossing rate, number of outcrossed fruit per tree, total number of fruit per tree, and tree yield declined slightly, but not significantly (*p* = 0.089, 0.088, 0.071, and 0.081, respectively) between the first and second ‘Shepard’ rows away from the interplanted ‘Hass’ rows. The percentage of ‘Shepard’ fruit that were fertilised by ‘Hass’ decreased significantly from the first to the second row away from ‘Hass’, whereas the percentage of ‘Shepard’ fruit that were fertilised by ‘Maluma’ increased significantly from the first to the second row away from ‘Hass’.

The ‘Shepard’ fruit that were fertilised by ‘Hass’ were 5% heavier than self-fertilised ‘Shepard’ fruit, without significant differences in diameter or length (Table 2). They had 24% heavier seeds than selfed fruit, without a significant difference in pericarp mass (Table 2). As a result, they had greater seed/total mass ratio than selfed fruit (Table 2).

## 3. Discussion

The study results confirmed our first hypothesis that outcrossing rates among avocado fruit would decline at increasing distances from blocks of trees of the alternate flowering type. This was particularly true in the single-cultivar ‘Maluma’ block, and it was true to some extent in the ‘Shepard’ block that contained interplanted rows of ‘Hass’ trees. The results also confirmed our second hypothesis that the number of cross-fertilised fruit per tree would decline at increasing distances from blocks of trees of the other cultivar. The results partly confirmed our third hypothesis that tree yields would decline at increasing distances from the other cultivar, with this being true in the ‘Shepard’ block. Tree yields did not decline in the middle of the ‘Maluma’ block, where much higher numbers of self-fertilised fruit were produced by each tree than in the ‘Shepard’ block. Finally, the results confirmed our fourth hypothesis that cross-fertilised fruit would be larger than self-fertilised fruit, although this depended on the cross-pollen parent.

The predominant visitors to avocado flowers at the study site included honeybees, consistent with reports from avocado orchards in many countries [59,60,62,71,80]. The other main flower visitors were syrphid flies. Flies have also been reported as major visitors to avocado flowers, and they can deposit pollen successfully on the stigma of avocado flowers [59,62,81,82,83,84]. Many flies carry and deposit fewer pollen grains than do honeybees [59,62] and they sometimes appear capable of supplementing rather than replacing the need for honeybee pollination [84]. Further research is required to determine whether flies forage over shorter distances than honeybees in orchards [85], potentially making them less frequent depositors of cross-pollen. The abundance of honeybees and syrphid flies, as well as other insects, was the same at different positions within the ‘Maluma’ and ‘Shepard’ blocks in the current study. This suggests that the reductions in outcrossing rates and the number of outcrossed fruit per tree at increasing distances from the neighbouring cultivar were related to differences in the pollen genotype carried by each pollinator rather than to the number of pollinators that visited each flower. The effective deposition of cross-pollen on flowers in single-cultivar blocks depends largely on the pollinators flying from female-phase flowers in a block of one cultivar to male-phase flowers in a block of a second cultivar. Our results confirmed a long overlap between the male-phase flowers of the Type B cultivar, ‘Shepard’, and the female-phase flowers of the Type A cultivar, ‘Maluma’. In addition, it is possible that some honeybees attain pollen by in-hive transfer from cultivars that they have not visited [86,87,88]. In either case, the effectiveness of each insect as a cross-pollinator also depends on the amount of cross-pollen carryover that occurs as it forages on successive flowers of the second cultivar [89,90,91]. Limited foraging ranges of pollinators in mass-flowering orchards [85,92,93,94], limited in-hive pollen transfer [86,87,88], and limited cross-pollen carryover during successive foraging bouts on flowers of the second cultivar [89,90,91] can all explain the lower levels of outcrossing encountered at increasing distances from polliniser trees in this and previous avocado studies [33,35,38,63,64,67].

The number of outcrossed fruit per tree declined less dramatically in the middle of the ‘Shepard’ block than the ‘Maluma’ block, largely as a result of the interplanting of ‘Hass’ polliniser trees in every fifth row of the ‘Shepard’ block. Three times as many fruit in the ‘Shepard’ block were cross-fertilised by ‘Hass’ than were cross-fertilised by ‘Maluma’. The ‘Shepard’ trees were 10–20 m away from ‘Hass’ trees within this block, whereas they were 10–90 m from ‘Maluma’ trees in the neighbouring block. Some of the ‘Shepard’ fruit that appeared to be fertilised by ‘Hass’ may, in fact, have been fertilised by ‘Carmen Hass’, as these two cultivars appear to be genetically identical [66]. However, the ‘Carmen Hass’ block was 80–210 m away from the sampled ‘Shepard’ trees in the study (Figure 4). The predominance of ‘Hass’ as a pollen parent in the ‘Shepard’ block demonstrates the value of closer interplanting of polliniser trees, including as single rows, for increasing the availability of cross-pollen in avocado orchards. However, there was still a decline in the number of outcrossed fruit per ‘Shepard’ tree at increasing distances from the neighbouring ‘Maluma’ block. The number of selfed fruit produced by each tree was relatively constant throughout the block, but yields declined by 25% at 60 m into the middle of the ‘Shepard’ block as a direct result of a 43% reduction in the number of outcrossed fruit per tree. These results are consistent with previous results from the adjoining ‘Hass’ block, where a 44% decline in yield at 160 m into the middle of the block was caused directly by a 69% reduction in the number of outcrossed fruit per tree rather than a difference in the number of selfed fruit per tree [67]. This shows that there was an underlying level of self-pollination through the orchard blocks but limited cross-pollination in the middle of the blocks. Outcrossing levels also decline at increasing distances from other cultivars in blocks of ‘Hass’, ‘Ettinger’, and ‘Fuerte’ in Israel, Spain, and the USA [33,35,38,63,64], with tree yields sometimes declining at 42–60 m into the middle of the blocks [33,35,63,64]. Recent results from orchards in Australia, Colombia, New Zealand, and Spain demonstrate that the stigmas of most avocado flowers receive very few or no pollen grains, so that most flowers are unpollinated or receive insufficient pollen grains to ensure fertilisation and fruit set [65,68,69,70]. Interplanting of different cultivars can increase the opportunities for successful pollen deposition, because the female-phase flowers of each cultivar potentially have closer access for many hours each day to cross-pollen from the male-phase flowers of an alternate-type cultivar [65,71,73]. Self-pollination, in contrast, relies on the female-phase flowers receiving pollen from male-phase flowers of the same cultivar during what is often a very limited period of synchronous opening each day [65,71,73].

However, outcrossing rates among avocado fruit are not always clearly related to tree yields [33,35,38,64], as observed among the ‘Maluma’ trees in the current study. The levels of outcrossing declined greatly in the middle of the ‘Maluma’ block but yields there were underpinned by the production of large numbers of self-fertilised fruit, almost twice as many per tree as in the middle of the ‘Shepard’ block. As a result, there was no evidence of pollen limitation to ‘Maluma’ yields, in contrast to the pollen limitation identified in the middle of the ‘Shepard’ block and previously in the middle of the adjoining ‘Hass’ block [67]. Many ‘Maluma’ trees contained both female-phase and male-phase flowers at the time of observations between 0900 H and 1300 H, when honeybees, syrphid flies, and other insects were active on the flowers. Female-phase ‘Maluma’ flowers may have had relatively long periods of access each day to self-pollen from male-phase flowers on the same or nearby ‘Maluma’ trees. Our results indicate that ‘Maluma’ trees experienced higher levels of self-pollination than did ‘Shepard’ trees, which could be confirmed by microscopic examination to assess whether more flowers in this block had pollen grains on their stigma or that each flower possessed a greater number of pollen grains on the stigma [65,68,69,70]. Trees in the middle of the ‘Maluma’ block were able to maintain high yields through a combination of both self-fertilised and cross-fertilised fruit production. Pollen limitation occurred in the ‘Shepard’ and ‘Hass’ blocks because low levels of self-pollination throughout the blocks could not be compensated adequately with cross-pollination in those trees that were planted in the middle of the blocks. ‘Maluma’ yields did not appear to be pollen-limited, indicating that fruit production was instead constrained by other factors. Avocado is a tropical species that is prone to water deficit and suboptimal yield in regions that have low rainfall, limited irrigation, and soils with low water-holding capacity [79,95,96,97]. Avocado yield can also be affected by insufficiency or over-supply of mineral nutrients, particularly nitrogen, phosphorus, potassium, boron, copper, magnesium, sulphur, and zinc [61,98,99,100]. Excessive nitrogen application can stimulate vegetative growth on avocado trees at the expense of flowering and fruit development, while nitrogen deficiency can reduce photosynthetic capacity. Fruit set and fruit retention are dependent on the tree producing photoassimilates and accumulating adequate carbohydrate reserves [101,102], including having high concentrations of starch in the flowers during pollination [103,104]. In the current study, the proportions of self-fertilised and cross-fertilised fruit that constituted tree yield changed throughout the ‘Maluma’ block. This suggests that outcrossed progeny were preferentially retained at the expense of selfed progeny when fruit load was higher than the physiological carrying capacity of the tree. Selective retention of outcrossed progeny has been demonstrated previously among ‘Hass’, ‘Ardith’, and ‘Ettinger’ avocado fruit [33,35,40], as well as among fruit of lychee (*Litchi chinensis* Sonn.), macadamia (*Macadamia integrifolia* Maiden & Betche and *M. tetraphylla* L.A.S.Johnson), and mango (*Mangifera indica* L.) [34,36,39].

Pollen-parent effects on fruit mass were evident in both cultivars, with cross-fertilisation by ‘Shepard’ increasing the mass of ‘Maluma’ fruit by 8%, and cross-fertilisation by ‘Hass’ increasing the mass of ‘Shepard’ fruit by 5%, when compared with self-fertilisation. These results are similar to findings for ‘Hass’ fruit, where cross-fertilisation by ‘Shepard’ increases fruit mass by 6–12% [40,67] and cross-fertilisation by ‘Fuerte’ increases fruit mass by 6% [69]. Our results confirm that positive xenia effects on avocado fruit size can occur in both directions, i.e., when a Type B cultivar is the pollen parent of the fruit of a Type A cultivar [40,67,69] and also when a Type A cultivar is the pollen parent of the fruit of a Type B cultivar [63]. Therefore, closer interplanting of a Type B polliniser such as ‘Shepard’ with Type A ‘Hass’ trees, for example, can increase the average size of both the ‘Shepard’ and the ‘Hass’ fruit. However, it appears that not all cross-pollen parents increase the size of the fruit, as cross-fertilisation by ‘Maluma’ did not significantly affect the mass of ‘Shepard’ fruit. Varying xenia effects among different cross-pollen parents are common among fruit and nut crops including apple [*Malus domestica* (Suckow) Borkh.], avocado, citrus, hazelnut (*Corylus avellana* L.), macadamia, plum, and strawberry [*Fragaria* × *ananassa* (Weston) Duchesne ex Rozier] [25,39,41,49,74,105,106,107].

Avocado growers often receive financial incentives for producing fruit of greater diameter [98,108,109,110,111] and so increasing the average fruit size by maximising the levels of cross-pollination has the potential to improve the profitability of orchards, not only through higher yields, but also through increasing the average fruit diameter. The results from the current study and previous studies in Australia, Israel, and the USA demonstrate that cross-pollination levels among fruit, and sometimes yields, are highest when avocado trees are planted in close proximity (often < 60 m) to trees of another cultivar [33,35,64,67]. However, the effects of poor pollination in orchards are often poorly understood by, or invisible to, growers. A grower does not see the fruit that failed to set when the flower was not pollinated. On the other hand, pests, diseases, mineral nutrient deficiencies, and water stress symptoms are often clearly visible, and the orchard management or financial costs associated with close interplanting of different cultivars, using narrow blocks or individual polliniser trees of each cultivar, are often better understood by growers. The alternative orchard design of planting wide blocks of each cultivar may allow pest, disease, irrigation, fertiliser, harvesting, and post-harvest processing to be more easily managed for individual cultivars [64,66,76,77,78]. For example, nitrogen application is recommended for ‘Hass’ trees during flowering, but it is preferable to avoid nitrogen application to ‘Shepard’ trees if they experience warm weather during flowering because high nitrogen levels can trigger excessive vegetative growth [79]. Also, ‘Shepard’ fruit reach maturity earlier than ‘Hass’ fruit and so separating the two cultivars into different blocks, rather than interplanting ‘Shepard’ polliniser trees within the ‘Hass’ rows, helps to ensure that each orchard row is traversed only once during harvesting and that fruit of these green-skinned and purple-skinned cultivars are not mixed together in the packing shed. Further research is needed to better understand the benefits and costs of interplanting different cultivars more closely to maximise fruit production and product quality versus establishing large blocks with many rows of each individual cultivar for cost-effective orchard and post-harvest management. This research may become more urgent as we experience growing shortfalls in the supply of managed pollinators for food production and a decline in the abundance and diversity of wild pollinators [14,112,113,114].

## 4. Materials and Methods

We sampled fruit in an avocado orchard (25°13′17″ S 152°18′45″ E) near Childers, Queensland, Australia, where outcrossing rates, yield, and fruit quality had been analysed 2 years earlier in a large block of ‘Hass’ trees [67]. This 95 ha orchard contained Type A cultivars ‘Hass’, ‘Maluma’, and ‘Carmen Hass’ in large single-cultivar blocks. It also contained two large blocks of Type B cultivar ‘Shepard’: one that was interplanted with a single row of ‘Hass’ trees every fifth row in the block, and one that was interplanted with a single row of ‘Maluma’ trees every fifth row in the block (see [67]).

We selected four ‘Maluma’ trees in each of 10 non-consecutive rows between the 7th and 22nd rows of a 26-row block of ‘Maluma’ trees (Figure 4). This block was surrounded on more than three sides by large blocks of ‘Hass’ and ‘Carmen Hass’, except that it adjoined the block of ‘Shepard’ and interplanted ‘Hass’ trees on its southern side (see [67]). We also selected four ‘Shepard’ trees in each of the 8th to 11th, 13th to 16th, and 18th to 21st rows of the 36-row block containing ‘Shepard’ and interplanted ‘Hass’ trees (Figure 4). This block adjoined the experimental ‘Maluma’ block on its northern side, a block of ‘Hass’ trees on its northeastern side, the block of ‘Shepard’ and interplanted ‘Maluma’ trees on its eastern side, and large blocks of ‘Hass’ and ‘Carmen Hass’ on its southern and western sides, respectively (see [67]). We selected the 2nd, 6th, 12th, and 23rd trees from the southern end of the 46-tree-long rows of ‘Maluma’ (Figure 4). The first tree at the southern end of each ‘Maluma’ row was immediately adjacent to the block of ‘Shepard’ and ‘Hass’ trees. We also selected the 2nd, 6th, 12th, and 18th trees from the northern end of the 35-tree-long rows of ‘Shepard’ (Figure 4). The first tree at the northern end of each ‘Shepard’ row was immediately adjacent to the block of ‘Maluma’ trees. The trees were 11 years old, and tree spacing was 10 m between rows and 5 m within rows. Therefore, the 2nd, 6th, 12th, and 23rd trees in each ‘Maluma’ row were 10 m, 30 m, 60 m, and 115 m, respectively, from the ‘Shepard’ trees, and the 2nd, 6th, 12th, and 18th trees in each ‘Shepard’ row were 10 m, 30 m, 60 m, and 90 m, respectively, from the ‘Maluma’ trees. The ‘Shepard’ trees were also either 10 m or 20 m from the interplanted rows of ‘Hass’ trees (Figure 4).

Eighty hives of honeybees and twelve hives of Australian stingless bees had been placed prior to flowering along the southern edge of the ‘Carmen Hass’ block and the block of ‘Shepard’ and ‘Hass’ trees, as well as at the northern end of the block of ‘Shepard’ and ‘Maluma’ trees (Figure 4). We counted insect visitors to flowers in the selected trees of six rows of each cultivar, ‘Maluma’ and ‘Shepard’, during peak flowering between 27 August 2023 and 13 September 2023. Insects were counted on four days for ‘Maluma’ and five days for ‘Shepard’. Visitor counts began at the commencement of anthesis and insect activity, between 0900 H and 1000 H, and continued until between 1200 H and 1300 H each day. We recorded all insect visitors in a 5 min period within a 1 m^3^ volume of the flowering canopy of each tree, randomising the order of observations for the trees within each row. Insects were considered flower visitors if they made contact with the anthers or stigma. Visitors were recorded as either honeybees, stingless bees, syrphid flies, or other visitors [61]. We also recorded whether each honeybee or stingless bee was foraging for nectar or pollen, and we recorded the functional gender of the flowers on each tree at the time of observation.

We subsequently hand-harvested ten mature fruit from each selected tree (i.e., 10 fruit × 88 trees = 880 fruit in total) using a stratified sampling design that divided each tree into four quadrants. Two quadrants were located on each side of the tree. We harvested two fruit per quadrant, one from the inside and one from the outside of the canopy, whenever possible. We also harvested two fruit near the tree trunk. ‘Shepard’ and ‘Maluma’ fruit were harvested at maturity on 27 February 2024 and 12 March 2024, respectively. We weighed the ten-fruit sample from each tree immediately after harvest and we counted all remaining fruit on the tree. Tree yield was calculated by multiplying the average fruit mass in the ten-fruit sample × the total number of fruit per tree. We then weighed each of the ten fruit in the sample individually and recorded their diameter and length. We allowed each fruit to ripen in the laboratory, we dissected the fruit, we weighed the seed and the pericarp, and we calculated the seed/total mass ratio.

DNA was extracted from the embryo in the seed of each of the 880 fruit using methods described previously [66]. We genotyped each embryo using the Agena MassARRAY platform (Agena Bioscience, San Diego, CA, USA) to assign paternity to each fruit. Paternity assignment was achieved by amplifying 34 DNA regions with unique single nucleotide polymorphisms (SNPs) that we identified from all four cultivars in the orchard as well as cultivars ‘Fuerte’, ‘Lamb Hass’, ‘Reed’, ‘Sharwil’, ‘Velvick’, and ‘Wurtz’. The primers used and the DNA sequences containing the SNPs have been described previously [40,66,115]. Cultivars ‘Hass’ and ‘Carmen Hass’ appear genetically identical [115] and were, therefore, considered as the same cultivar when assigning fruit paternity.

We calculated the proportion of harvested fruit that were outcrossed in each ‘Maluma’ or ‘Shepard’ tree. We estimated the total number of outcrossed fruit per tree by multiplying the proportion of harvested fruit that were outcrossed × the total number of fruit per tree. We calculated the number of selfed fruit per tree by subtracting the number of outcrossed fruit per tree from the total number of fruit per tree. In addition, we calculated the proportion of fruit that were fertilised by ‘Hass’ and the proportion of fruit that were fertilised by ‘Maluma’ in each ‘Shepard’ tree.

We assessed the effect of distance to the neighbouring orchard block on the numbers of honeybees, stingless bees, syrphid flies, and other insects visiting avocado flowers using Kruskal–Wallis and median tests. We assessed the effect of distance to cultivar ‘Shepard’ on the outcrossing rate, fruit number, and yield of ‘Maluma’ trees using random block analysis of variance (ANOVA), regarding the 10 different orchard rows of ‘Maluma’ as blocks. We assessed the effects of distance to cultivar ‘Maluma’ and distance to cultivar ‘Hass’ on the outcrossing rate, ‘Maluma’ parentage rate, ‘Hass’ parentage rate, fruit number, and yield of ‘Shepard’ trees using two-way ANOVA, with each pair of ‘Shepard’ orchard rows adjacent to a ‘Hass’ row being incorporated as a block in the analysis. Tukey’s Honestly Significant Difference (HSD) tests were performed when differences among four distance means were detected by ANOVA. We assessed the effect of pollen parentage on fruit diameter, fruit length, total fruit mass, seed mass, pericarp mass, and seed/total mass ratio by two-way or three-way ANOVA, with pollen parent and distance to the polliniser(s) incorporated as factors and orchard row incorporated as a block. Tukey’s Honestly Significant Difference (HSD) tests were performed when differences among three pollen-parent means were detected by ANOVA. Means, medians, and data distributions were regarded as significantly different at *p* < 0.05. Means are reported with standard errors (SEs). Medians are presented with 25th and 75th percentiles, 10th and 90th percentiles, and outliers.

## 5. Conclusions

The number of cross-fertilised fruit produced by each avocado tree declined in the middle of large, adjoining, single-cultivar blocks of ‘Maluma’ and ‘Shepard’. Yields in the ‘Maluma’ block did not appear to be pollen-limited, as trees in the middle of the block could compensate for lower levels of cross-fertilisation by producing greater numbers of self-fertilised fruit. However, yields in the middle of the ‘Shepard’ block were pollen-limited. The number of self-fertilised fruit produced by each tree was low throughout the ‘Shepard’ block, and yield declined by 25% in the middle of the block because of a 43% reduction in the number of cross-fertilised fruit per tree. There, the combination of low self-pollination and low cross-pollination was insufficient to generate high yields. Furthermore, cross-fertilisation by ‘Shepard’ increased the mass of ‘Maluma’ fruit by 8%, and cross-fertilisation by ‘Hass’ increased the mass of ‘Shepard’ fruit by 5%, when compared with self-fertilisation. Outcrossing rates are sometimes, but not always, related to tree yields in avocado orchards. Our results indicate that the effect on yield of increasing the level of cross-pollination may often depend on whether or not there are sufficient levels of self-pollination to produce high yields. In either case, higher levels of cross-pollination can increase the average fruit mass, and avocado growers are often paid higher prices for larger fruit. Further research is warranted to better understand the benefits of interplanting different cultivars more closely to maximise fruit production and quality, especially as food producers adapt to global shortfalls in the availability and diversity of insect pollinators.

## Figures and Tables

**Table 1:** Diameter, length, total mass, seed mass, pericarp mass, and seed/total mass ratio of selfed and outcrossed ‘Maluma’ avocado (*Persea americana* Mill.) fruit.

Parameter	Mother Cultivar × Father Cultivar	
	‘Maluma’ × ‘Maluma’ (Selfed)	‘Maluma’ × ‘Shepard’ (Outcrossed)
Diameter (mm)	66.6 ± 0.3 a	68.6 ± 0.5 b
Length (mm)	100.0 ± 0.6 a	101.2 ± 0.9 b
Total fruit mass (g)	215.7 ± 2.8 a	233.8 ± 4.6 b
Seed mass (g)	19.8 ± 0.5 a	21.8 ± 0.7 b
Pericarp mass (g)	196.0 ± 2.4 a	212.1 ± 4.0 b
Seed/total mass (%)	8.9 ± 0.1 a	9.1 ± 0.2 a

Means (±SE) with different letters within a row are significantly different (two-way ANOVA; *p* < 0.05; n = 246 selfed fruit and 144 outcrossed fruit).

**Table 2:** Diameter, length, total mass, seed mass, pericarp mass, and seed/total mass ratio of selfed and outcrossed ‘Shepard’ avocado (*Persea americana* Mill.) fruit.

Parameter	Mother Cultivar × Father Cultivar		
	‘Shepard’ × ‘Shepard’ (Selfed)	‘Shepard’ × ‘Maluma’ (Outcrossed)	‘Shepard’ × ‘Hass’ (Outcrossed)
Diameter (mm)	63.9 ± 0.2 a	63.6 ± 1.2 a	65.3 ± 0.6 a
Length (mm)	106.2 ± 0.4 a	103.4 ± 1.8 a	104.8 ± 1.1 a
Total fruit mass (g)	202.8 ± 1.7 a	195.4 ± 8.9 a	213.1 ± 4.5 b
Seed mass (g)	35.5 ± 0.5 a	36.6 ± 2.5 a	44.1 ± 1.3 b
Pericarp mass (g)	167.3 ± 1.4 a	158.8 ± 6.8 a	169.0 ± 3.5 a
Seed/total mass (%)	17.4 ± 0.2 a	18.4 ± 0.7 a	20.6 ± 0.4 b

Means (±SE) with different letters within a row are significantly different (three-way ANOVA and Tukey’s HSD test; *p* < 0.05; n = 25–323 fruit).

## Data Availability

The data presented in this study are available upon request from the corresponding author and with the permission of Hort Innovation.
